# Tadalafil Rescues the p.M325T Mutant of Best1 Chloride Channel

**DOI:** 10.3390/molecules28083317

**Published:** 2023-04-08

**Authors:** Kathleen Elverson, Jim Warwicker, Sally Freeman, Forbes Manson

**Affiliations:** 1 Division of Evolution, Infection and Genomics, Faculty of Biology, Medicine and Health, The University of Manchester, Manchester M13 9PT, UK; 2 Division of Molecular and Cellular Function, Faculty of Biology, Medicine and Health, Manchester Institute of Biotechnology, The University of Manchester, Manchester M1 7DN, UK; 3 Division of Pharmacy and Optometry, School of Health Sciences, Faculty of Biology, Medicine and Health, The University of Manchester, Manchester M13 9PT, UK

**Keywords:** bestrophin, IRDs, virtual docking, FDA-approved, COPII, 4PBA, tadalafil, whole-cell patch-clamp

## Abstract

Bestrophin 1 (Best1) is a chloride channel that localises to the plasma membrane of retinal pigment epithelium (RPE) cells. Mutations in the BEST1 gene are associated with a group of untreatable inherited retinal dystrophies (IRDs) called bestrophinopathies, caused by protein instability and loss-of-function of the Best1 protein. 4PBA and 2-NOAA have been shown to rescue the function, expression, and localisation of Best1 mutants; however, it is of interest to find more potent analogues as the concentration of the drugs required is too high (2.5 mM) to be given therapeutically. A virtual docking model of the COPII Sec24a site, where 4PBA has been shown to bind, was generated and a library of 1416 FDA-approved compounds was screened at the site. The top binding compounds were tested in vitro in whole-cell patch-clamp experiments of HEK293T cells expressing mutant Best1. The application of 25 μM tadalafil resulted in full rescue of Cl^−^ conductance, comparable to wild type Best1 levels, for p.M325T mutant Best1 but not for p.R141H or p.L234V mutants.

## 1. Introduction

Bestrophin 1 (Best1) is a Ca^2+^- dependent Cl^−^ channel that primarily localises to the basolateral plasma membrane of retinal pigment epithelium (RPE) cells [1,2]. Mutations in the *BEST1* gene are associated with a group of inherited retinal dystrophies (IRDs) called bestrophinopathies, caused by protein instability and loss-of-function of the Best1 protein. Over 350 mutations in *BEST1* have been identified and result in clinically distinct diseases including Best vitelliform macular dystrophy (BVMD), autosomal recessive Bestrophinopathy (ARB), autosomal dominant vitreoretinochoroidopathy (ADVIRC) and retinitis pigmentosa (RP) [3,4]. Bestrophinopathies cause blindness and are currently incurable.

Research on different strategies for the treatment of bestrophinopathies and other IRDs has mainly focused on genome editing and gene- and cell-based therapy due to recent advances in molecular genetics and the immune privilege of the human eye [5]. However, there have also been developments in pharmacological approaches, for example, in the use of chemical chaperones to correct protein folding of mutant proteins causing RP, or drugs that target the visual cycle as a treatment for Stargardt disease [6,7].

**Figure 1:**
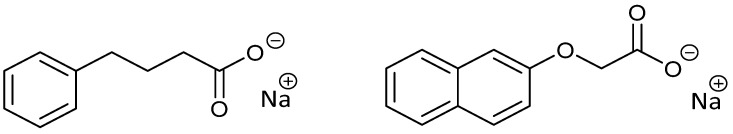
Sodium salt of 4-Phenylbutyric acid (4PBA) and 2-naphthoxyacetic acid (2-NOAA).

The small molecule sodium phenylbutyrate (4PBA) and its analogue 2-naphthoxyacetic acid (2-NOAA) (Figure 1) can restore the expression, localisation, and function of mutant Best1 that causes ARB and BVMD [8,9]. The concentrations of 4PBA and 2-NOAA are too high (~2.5 mM) to be used therapeutically [10]; therefore, it is of interest to find more potent drugs as potential treatments.

4PBA is an FDA- and EMA-approved orally administrated drug for the treatment of urea cycle disorders [11]. It has a secondary function in increasing the function of missense proteins although the mode of action is unknown. It has been suggested to act as a chemical chaperone for the treatment of cystic fibrosis [12], cerebral ischemia [13] and congenital nephrotic syndrome [14]. 4PBA has been shown to induce the synthesis of molecular chaperones and downregulate protein synthesis in vivo [10,15]. The regulation of genes involved in the unfolded protein response (UPR) pathway is characteristic of histone deacetylase inhibitors (HDACis) and the potential anticancer properties of 4PBA via its HDACi activity on cell proliferation is under investigation [16,17,18].

4PBA has also been suggested to act as a proteostasis regulator through attenuation of endoplasmic reticulum (ER) stress. Work by Ma et al. (2017) showed that 4PBA competes with the binding of p24 proteins in the formation of COPII (Coat Protein Complex II) vesicles [19]. Crystallographic analysis of the Sec24a binding site on COPII showed evidence of 4PBA binding at low concentrations. The structure of 4PBA closely resembles the ΦC ER (C-terminal aromatic endoplasmic reticulum) export signal motif which binds at the Sec24a site, identified in studies of the p24–family proteins. Therefore, 4PBA may promote the packaging of mutant proteins into COPII vesicles and their anterograde transport to the Golgi apparatus, reducing the stringency of their retention in the ER [19].

Virtual screening (VS) is often employed in the early stages of drug discovery as a low cost, effective technique for identifying potential drug leads [20]. The process involves the in silico docking of a large library of compounds into the binding site of a protein, allowing the potential differentiation between active and inactive compounds for biological testing [21]. As 4PBA binds to COPII, the first objective of this work was to establish and validate a docking model of the Sec24a COPII binding site.

Molecular docking software predicts the non-covalent binding interactions between small molecule ligands and the macromolecular receptor protein [22]. The molecular conformation of a ligand in the receptor binding pocket is evaluated and its binding affinity to the receptor is then calculated through the use of a scoring function [23]. Although there are a number of docking software packages, most show similar results when tested on a series of protein-ligand complexes [24]. In this research, AutoDock Vina was used, a freely available software that employs a Monte Carlo-based search algorithm and an empirical scoring function [22].

In this study, we docked 1416 compounds from an FDA-approved drug library into the Sec24a COPII binding site. The top binding drugs were identified after post docking filtering for in vitro testing by whole-cell patch-clamp, the gold standard technique for measuring ion channel conductance across the cell membrane [25]. Compounds were tested for their ability to restore the Cl^−^ conductance of Best1 mutants (p.L234V associated with BVMD and p.M325T associated with ARB) at 100-fold lower concentrations (25 µM) than 4PBA to identify more effective drugs for the treatment of the bestrophinopathies.

## 2. Results

### 2.1. Generation of the COPII Sec24a Docking Model

**Figure 2:**
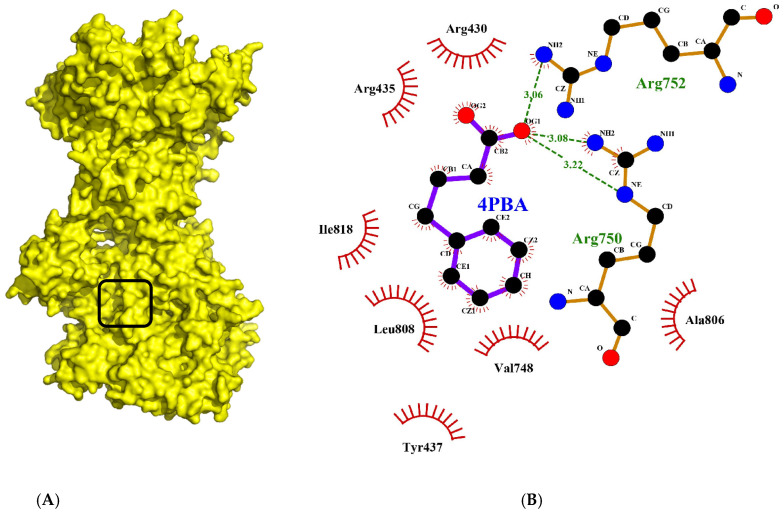
(**A**) COPII protein (pdb 5vnl) with the Sec24a binding site highlighted, figure generated from pdb file 5vnl using PyMOL 1.3 software [19,26]. (**B**) Interactions between the 4PBA conformer obtained from docking studies with residues in the COPII Sec24a binding site are shown. Hydrophobic contacts (represented by red arcs and spokes) are shown between the 4PBA benzyl group and Val748, Leu808, Ile818, and Tyr437 residues. The 4PBA carboxylate head group forms key hydrogen bond interactions (represented by green dashes) with residues Arg750 and Arg752 and hydrophobic contacts with Arg435 and Arg430. Diagram produced with LigPlot+ from the docked 4PBA conformer and pdb 5vnl [19,27].

A docking model of COPII was first generated with the objective of screening a large library of molecules in the Sec24a 4PBA binding site (Figure 2A) in order to find compounds that bind with a higher affinity than 4PBA. The docking model was validated in re-docking experiments with 4PBA using AutoDock Vina 1.1.2 software [22] (Supplementary Materials S1).

The 4PBA ligand was docked into the binding site as the carboxylate anion to give a binding energy (BE) of −5.8 kcal mol^−1^, forming several key interactions with residues in the binding site (Figure 2B), including hydrophobic contacts between the phenyl group with V748, L808, I818, and Y437. The carboxylate anion head group formed moderately strong, mostly electrostatic hydrogen bonding with the positively charged guanidino side chains of residues R750 and R752 and hydrophobic interactions with R435 and R430.

### 2.2. Screening of an FDA-Approved Drug Library into the COPII Sec24a Docking Model

A library of 1416 FDA-approved compounds from the DrugBank database [28] was screened into the docking model and the strongest BE for each compound was collated and analysed. Post docking filtering was applied using the ChemBioServer, a publicly available online application used to increase the efficiency of post process drug candidate selection in virtual screening studies of the PI3Kα protein [29,30]. The results were filtered based on Lipinski’s rules (Mr < 500 g mol^−1^, hydrogen bond donors ≤ 5, hydrogen bond acceptors ≤ 10, partition coefficient (log P) ≤ 5) and Veber’s Rules (polar surface area ≤ 140 Å, rotatable bonds ≤ 10) as these are good measures for the potential oral bioavailability of a compound [31,32]. This filter ruled out 312 compounds. The remaining 1104 compounds were ranked by binding energy; the top five candidates from the library (Table 1) range in binding energies from −9.1 to −8.8 kcal mol^−1^, three orders of magnitude stronger binding than 4PBA at −5.8 kcal mol^−1^. Here, binding energy (BE) is used as a ranking to identify more potent compounds than 4PBA, not as an absolute free energy measurement (the individual terms in AutoDock Vina’s scoring function are provided as relative scores) [22,33].

The six compounds with the highest BEs are shown in Table 1. Lumacaftor is a chemical chaperone that improves the processing of mutant ΔF508 cystic fibrosis transmembrane conductance regulator (CFTR) and its transport to the cell surface [34]. It is of note that Lumacaftor has been shown to rescue the processing and cell surface expression of ABCA4 mutants associated with Stargardt Disease [35]; however, by western blot we previously found that it did not increase the expression of mutant Best1.

**Table 1:** Top hits from docking studies of the DrugBank FDA library in the Sec24a site of COPII. Compounds were docked as anions where appropriate, the best binding pose and corresponding binding energy for each compound is displayed. LogP values are computed by XLogP3 3.0 on PubChem database [36], binding pose images generated in PyMOL with pdb 5vnl [19,26].

Name ZINC ID	Structure	Best Binding Pose	Binding Energy/kcal mol^−1^	M_r/_ g mol^−1^	LogP
Lumacaftor ZINC000064033452	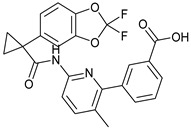	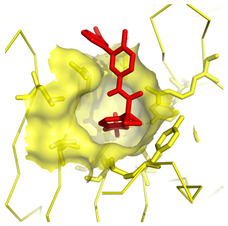	−9.1	452	4.4
Risperdal ZINC000000538312	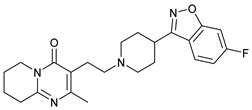	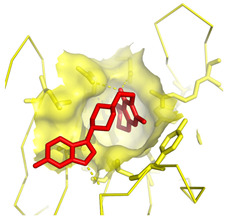	−9.0	411	2.7
Lurasidone ZINC000003927822	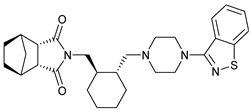	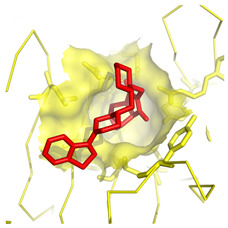	−8.9	493	5.4
Thalidomide ZINC000001530948	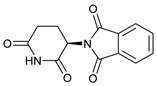	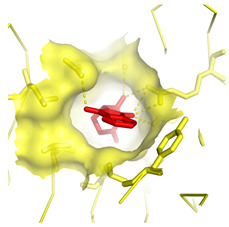	−8.8	258	0.3
Tadalafil ZINC000003993855	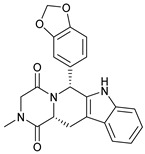	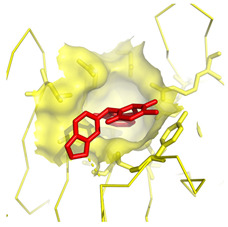	−8.8	389	2.3
Paliperidone ZINC000004214700	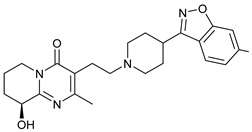	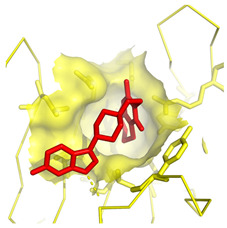	−8.8	427	2.2
4-Phenylbutanoic acid	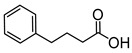	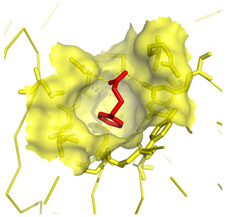	−5.8	164	2.4

### 2.3. Tadalafil Forms Key Hydrophobic Interactions in COPII Sec24a Site

**Figure 3:**
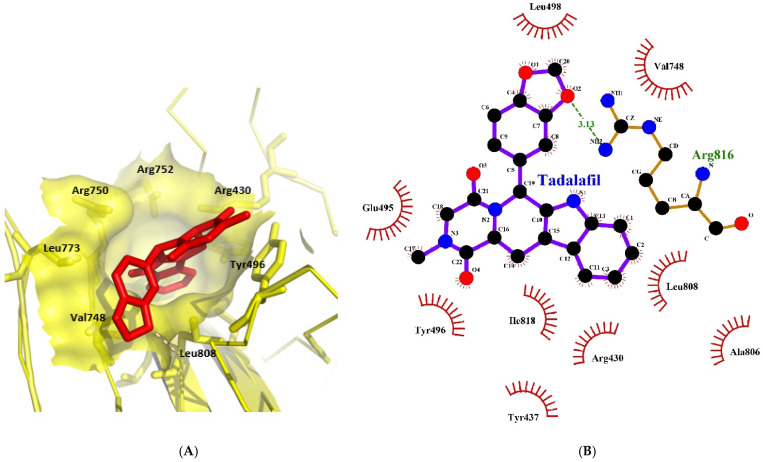
Interaction of tadalafil with COPII binding site. (**A**) The tadalafil ligand (red) docked into in the Sec24a binding site of COPII (pdb 5vnl) is shown with the benzodioxole group lying on the adjacent shelf region. (**B**) Interactions between the tadalafil conformer with residues in the COPII binding site are shown. Hydrophobic contacts (represented by red arcs and spokes) are shown for Val748, Leu808, Ala806, Arg430, Tyr437, Ile818, Tyr496, and Glu495 residues. The ether oxygen of tadalafil also forms a hydrogen bond interaction (represented by green dashes) with Arg816. Diagram produced with LigPlot+ from the docked tadalafil conformer and pdb 5vnl [19,27].

Tadalafil was docked into the Sec24a binding site of COPII and formed a large number of hydrophobic contacts with residues in the binding pocket (Val748, Leu808, Ala806, Arg430, Tyr437, Ile818, Tyr496, and Glu495), and a hydrogen bond interaction between its benzodioxole group and Arg816 (Figure 3A,B). The large number of interactions made by tadalafil contributes to a three-fold greater binding energy than 4PBA in the COPII site. This docking shows Tadalafil binding close to the 4PBA site in COPII, but with higher affinity than 4PBA.

### 2.4. Tadalafil Rescues Cl^−^ Conductance of p.M325T ARB Mutant hBest1

**Figure 4:**
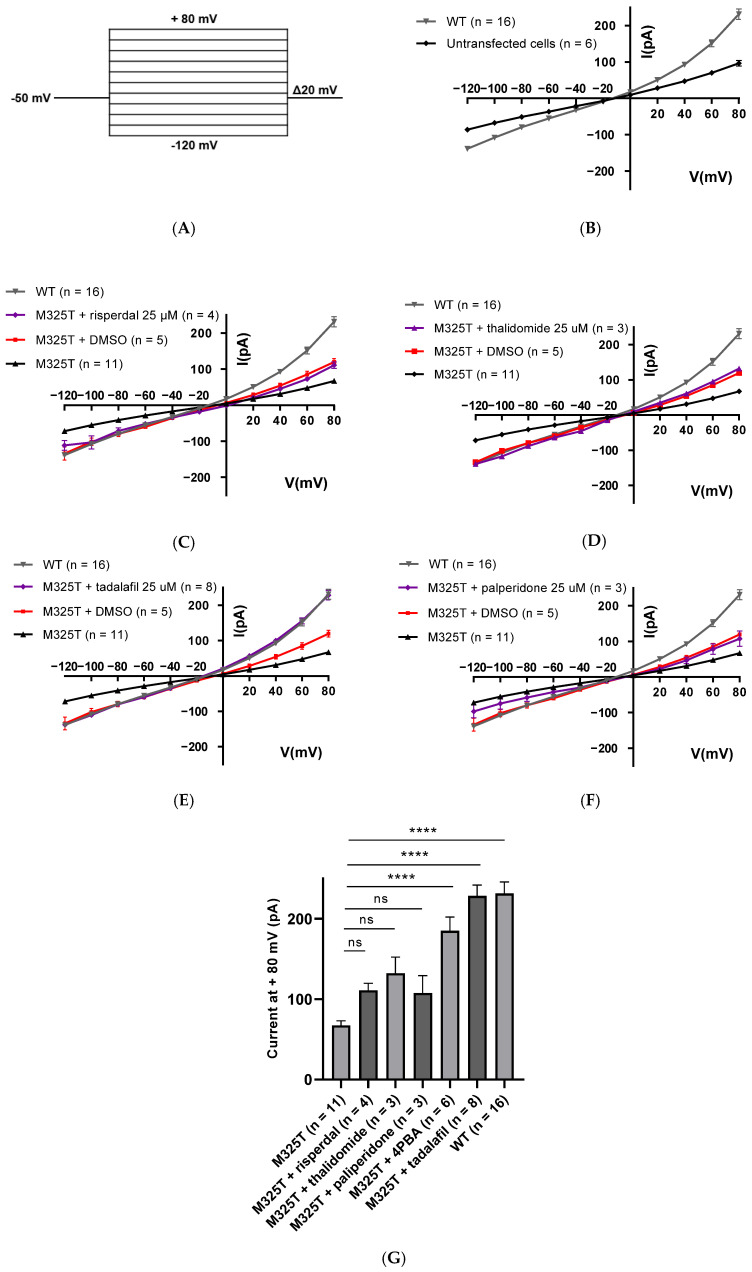
Effect of drugs on M325T Best1 Cl^−^ currents recorded in HEK293T cells by whole-cell patch-clamp experiments. (**A**) Voltage protocol; the membrane potential was held at −50 mV and recordings were taken in the range of −120 mV to +80 mV in Δ20 mV steps of 2 s each. (**B**) Mean current/voltage relationships for wild type (WT) Best1 and untransfected HEK293T cells. (**C**–**F**) Mean current/voltage relationships for p.M325T Best1 expressing cells before and after treatment with 25 µM risperdal, thalidomide, tadalafil and paliperidone, respectively. (**G**) Currents measured at +80 mV (pA) for p.M325T Best1 before and after treatment with drugs. Addition of 25 µM tadalafil results in full rescue of p.M325T Cl^−^ conductance to WT levels (results presented as mean ± s.e.m, **** *p* < 0.0001, as calculated by one-way ANOVA). p.M325T + 2.5 mM 4PBA (dissolved in water) is shown for reference.

**Figure 5:**
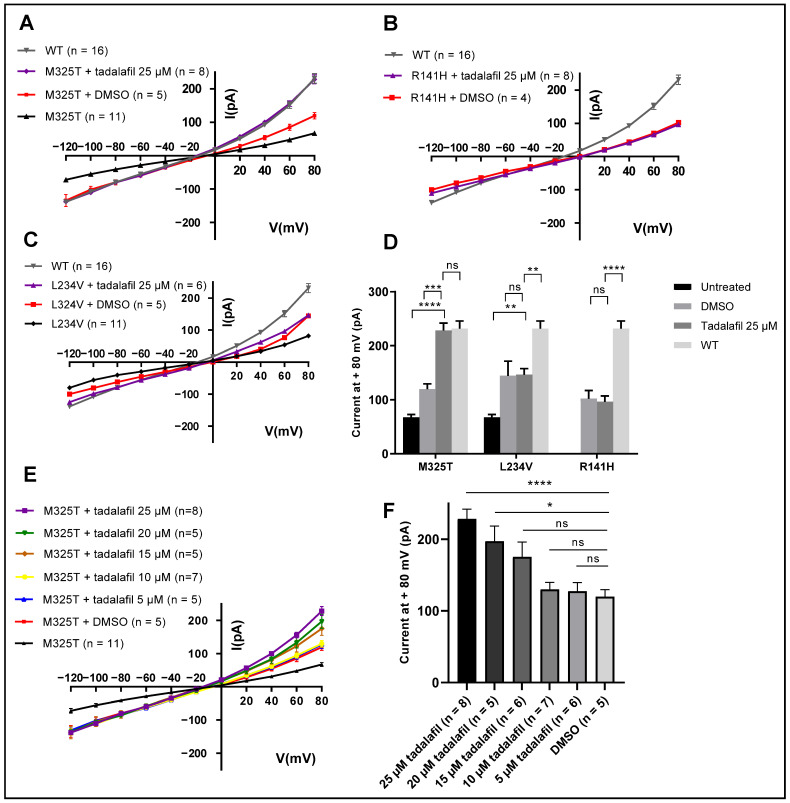
Effect of tadalafil on mutant Best1 Cl^−^ currents recorded in HEK293T cells by whole-cell patch-clamp experiments. The membrane potential was held at −50 mV and recordings were taken in the range of −120 mV to +80 mV in Δ20 mV steps of 2 s each. (**A**–**C**) Mean current/voltage relationships for mutant Best1 expressing cells (p.M325T, p.R141H (ARB) and p.L234V (BVMD)) before and after treatment with 25 µM tadalafil. (**D**) Currents measured at +80 mV (pA) for mutant Best1 before and after treatment with 25 µM tadalafil. Tadalafil fully rescues the Cl^−^ conductance of p.M325T to WT levels but not for p.R141H or p.L324V mutants (results presented as mean ± s.e.m, ** *p* < 0.01, *** *p* < 0.001, **** *p* < 0.0001, as calculated by one-way ANOVA). (**E**) Dose response effect shown for Cl^−^ current of p.M325T Best1 expressing cells treated with decreasing concentrations of tadalafil. (**F**) Currents measured at +80 mV (pA) for p.M325T Best1 expressing cells treated with decreasing concentrations of tadalafil (Results presented as mean ± s.e.m, * *p* < 0.05, **** *p* < 0.0001, as calculated by one-way ANOVA).

The top five binding compounds from the FDA screen (risperdal, thalidomide, tadalafil, paliperidone, and lurasidone) were then tested by whole cell patch-clamp recording, a technique which measures the membrane current of an entire cell by forming a seal with the membrane. Control patch-clamp experiments are shown in the Supplementary Materials (Figures S3 and S4), confirming that cells expressed a Ca^2+^-activated Cl^−^ channel. HEK293T cells expressing mutant ARB (p.M325T) hBest1 were treated with the compounds at 25 µM for 24 h (Figure 4). The application of 25 µM of Lurasidone was cytotoxic to HEK293Ts so was excluded from further study. Treatment with 25 µM of tadalafil, a known phosphodiesterase-5 (PDE5) inhibitor, showed significant rescue of M325T Cl^−^ conductance, comparable to wild type Best1 levels, with a strong dose response effect (Figure 5) (raw data are shown in Figure S5) [37]. The docking shown in Section 2.3 is consistent with the enhanced ability of tadalafil to rescue the p.M325T mutant of Best1 chloride channel, when compared with 4PBA. However, no rescue was shown for cells expressing p.R141H (associated with ARB) or p.L234V (associated with BVMD) mutant Best1 (Figure 5).

## 3. Discussion

Repurposing previously approved drugs has several distinct advantages over the development of novel drugs; the safety of the compound will have been established through toxicity tests and pharmacokinetic/pharmacodynamic (PK/PD) studies, therefore bypassing the need for Phase 1 clinical trials. Research and development costs and the time needed to bring the drug to market are also considerably cut [38].

In this study, we sought to find more potent drugs for treatment of the bestrophinopathies by virtually screening a library of FDA compounds into a model of the COPII Sec 24a binding site, where 4PBA, a rescuer of mutant Best1 function, has been shown to bind [9,19]. The computational model was successfully validated following re-docking experiments with 4PBA, followed by the docking of a library of 1416 compounds into the binding site to identify the top binding compounds. There are a few limitations to this docking model; AutoDock Vina makes the assumption that the receptor is rigid, keeping covalent lengths and angles constant, whilst allowing rotation of ligand molecules (known as ‘flexible docking’) [22]. In reality, the COPII protein will show a high degree of conformational flexibility which may lead to errors in calculated binding energies. The docking model is also dependent on the mechanism of 4PBA action. It is unknown if 4PBA acts as a chemical chaperone, HDACi, a proteostasis regulator through binding to COPII vesicles, or, most likely, through a combination of these pathways. Therefore, compounds that have a higher affinity for the COPII site than 4PBA may not necessarily be more potent rescuers of mutant Best1 function; however, virtual screening into the COPII site is a rational method for generating potential 4PBA analogues.

We selected the 5 top binding compounds to test in whole-cell patch-clamp experiments. The small molecule tadalafil was able to increase p.M325T ARB Best1 function to wild type level at 25 µM. In contrast, the other compounds (risperdal, thalidomide, and paliperidone) showed no effect at 25 µM. This is a 100-fold-improvement on the potency of 4PBA which is active at 2.5 mM. Surprisingly, tadalafil had no effect on the Cl^−^ conductance of another ARB mutant, p.R141H, or the BVMD mutant, p.L234V, at 25 µM.

Tadalafil was originally developed as a treatment for erectile dysfunction in addition to other PDE5 inhibitors: sildenafil, vardenafil, and avanafil [39]. This class of compounds has been the focus of recent research for other purposes, such as to increase bone mass [40], treat pulmonary hypertension [41,42], and act as anticancer agents [43]. The inhibition of PDE5 causes an increase in cyclic-guanosine monophosphate (cGMP) levels, a regulator of CFTR expression. Tadalafil, sildenafil, and vardenafil were successful in restoring the Cl^−^ transport of ΔF508 mutant CFTR proteins across the nasal mucosa of mice after intraperitoneal injection [44]. Recent studies showed that vardenafil was able to promote the early steps of cellular processing and trafficking of F508del CFTR through a cGMP-independent mechanism in human bronchial epithelial cells [45].

The predominant form of PDE in the retina is PDE6, which is found in the internal membranes of rod and cone photoreceptors and regulates cGMP levels in response to light [46]. There have been some studies that suggest PDE5 inhibitors cause side effects in the eye, for example, non-arteritic ischemic neuropathy from sildenafil use; however, more studies are needed to determine if there is a cause-and-effect relationship [47]. Tadalafil has a weaker effect on visual function than sildenafil, which may relate to its high PDE5 selectivity over PDE6 [48]. The link between Best1 and PDE is unknown; we propose that tadalafil may act as a proteostasis regulator by binding to COPII and reducing the retention of misfolded Best1 in the ER to restore mutant function.

**Figure 6:**
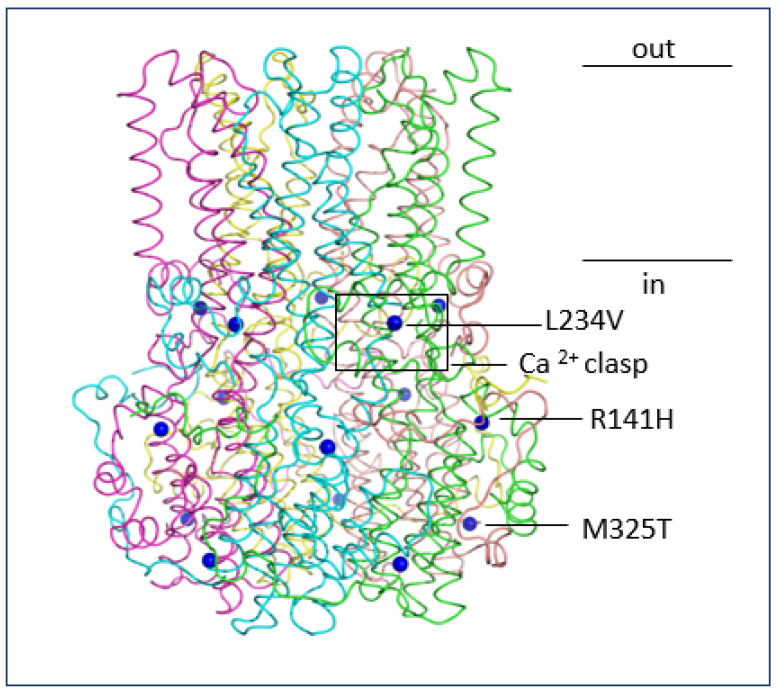
L234V, R141H and M325T missense mutation locations in a model of hBest1. Alpha carbon atoms of L234V, R141H and M325T missense mutations (from the HGMD) are displayed on each monomer of the hBest1 homopentamer model as blue spheres. The tube backbone of modelled hBest1 is colour-coded by chain. The location of a calcium clasp and approximate boundaries of the transmembrane region are marked, together with indication of inside or outside of cell.

It is curious why tadalafil increases the function of mutant p.M235T Best1 but not the p.R141H (ARB) or p.L234V (BVMD) mutants. 4PBA fully rescues both p.R141H and p.M325T and partially rescues p.L234V mutants [8,9]. p.M325T is located on a portion of the intracellular C-terminal tail which has not yet proven to be functionally significant (auto-inhibitory segment is formed from residues 346–379), in comparison to p.R141H and p.L234V, which are closer to functionally important sites (Figure 6). p.L234V is located at a site that forms key interactions with an acidic residue cluster, close to the Ca^2+^ clasp site [49]. Therefore, due to its disruption of the Ca^2+^ active site, the p.L234V mutant may be less active than the p.M325T mutant and its function harder to rescue. p.R141H is located in a cytosolic loop between two transmembrane domains and may disrupt Best1 regulation [50]. It is possible that the Cl^−^ activity of p.M325T Best1 is easier to rescue as the mutant is less damaging to overall protein function. However, more research is needed to determine the number of Best1 mutants, which show a restoration of Cl^−^ conductance when treated with tadalafil in comparison to 4PBA.

The repurposing of drugs is a relatively quick and economic route to drug discovery for new conditions. Here, we have demonstrated that tadalafil can fully restore the function of a mutant Best1 associated with a rare disease that lacks any therapeutic treatment options. Further investigation is required to determine whether tadalafil has the potential as a therapeutic treatment for bestrophinopathies, but nonetheless, it is a promising lead compound which can inspire the future design and development of therapeutic small molecules for this currently untreatable genetic eye disorder.

## 4. Materials and Methods

### 4.1. Virtual Docking Studies of COPII Binding Site

#### 4.1.1. Validation of Docking Model

Electron density files (pdb 5vnl, 5vnn) for the COPII Sec24a binding site were viewed in Coot [19,51]. A receptor pdbqt file of COPII (pdb 5vnl, resolution 2.4 Å) was generated using AutoDock Tools [52]. Crystallographic water molecules were left in, and hydrogen atoms were added to the structure. Gasteiger charges were added, and non-polar hydrogen atoms were merged. The coordinates of the COPII Sec24a binding site were identified and the search space (15 × 15 × 15 Å) was set by calculating the centre of mass of key binding site residues.

The ligand pdbqt file of 4PBA was generated by extracting the 4PBA ligand from pdb 5vnl using PyMOL [26]. The torsion root of 4PBA was then detected in AutoDock Tools and rotatable bonds were set. Gasteiger charges were added, and non-polar hydrogen atoms were merged. AutoDock Vina was run for the ligand and receptor files with an exhaustiveness of 16 [22]. The ligand output pdbqt file yielded 9 conformers, which were viewed and analysed in PyMOL; the best conformer with the lowest binding energy was selected. RMSD values were calculated in PyMOL.

#### 4.1.2. Docking of DrugBank FDA Libraries

Ligand pdbqt files for the DrugBank FDA library [28] were generated by conversion of mol2 files (available on ZINC website [53]) to pdbqt format using OpenBabel 3.1.1 software [54]. Ligands were docked using AutoDock Vina on the Computational Shared Facility 3 Manchester (CSF3) and results were viewed in PyMOL. The lowest binding energy of each conformer was collated and analysed in Excel.

Post docking filtering was applied to the FDA library based on Lipinski’s and Veber’s rules using the ChemBioServer [29]. Properties of selected compounds (Table 1) were obtained from ZINC and PubChem databases. Statistical analysis was performed by Pearson correlation coefficient using GraphPad Prism version 9.1.2 for Windows, GraphPad Software, San Diego, CA, USA, www.graphpad.com (accessed on 30 June 2022).

### 4.2. Cell Culture

Human Embryo Kidney 293T (HEK293T) cell lines were cultured using Dulbecco’s Modified Eagle’s Medium (high glucose) supplemented with 10% heat inactivated foetal bovine serum (FBS) and 1% L-glutamine (L-glut). Cell lines were incubated in a humidified atmosphere at 37 °C, 5% CO_2_, and passaged when cells reached 70% confluence.

### 4.3. Plasmid Extraction

Plasmid DNA was extracted from overnight cultures using the QIAprep miniprep kit^®^ (Qiagen) according to the manufacturer’s instructions. Plasmid concentrations were determined using a NanoPhotometer N60 spectrophotometer (GeneFlow Ltd., Lichfield, UK).

### 4.4. Whole-Cell Patch-Clamp

#### 4.4.1. Transient Transfection

HEK293T cells were co-transfected with wild type or mutant Best1 and GFP in a 4:1 ratio using Fugene HD transfection reagent (Promega, Madison, WI, USA) in a reverse or normal transfection protocol. Cells were seeded on 13 mm glass coverslips treated with 0.01% poly-L-lysine solution (P4832, Sigma-Aldrich, St. Louis, MO, USA) at least 24 h prior to patch-clamp experiments.

#### 4.4.2. Small Molecule Treatment

Compounds were added to cell media 24 h prior to patch-clamp experiments. 4PBA (1716-12-7, Sigma-Aldrich) was dissolved in water at a concentration of 2.5 mM. All other compounds were dissolved in water and DMSO (0.001% final concentration in media).

### 4.5. Whole-Cell Patch-Clamp Recordings

Whole-cell voltage-clamp recordings were performed using borosilicate glass capillaries GC100F-10 (Harvard Apparatus, Edenbridge, UK). Pipettes were pulled with a Model P-97 pipette puller (Sutter Instrument Co., Novato, CA, USA) and fire-polished to a resistance of 1–2 MΩ. The electrophysiology setup consisted of a MultiClamp 700A amplifier controlled by pCLAMP 10.4 (Molecular Devices, Wokingham, UK). Cell membrane potential was held at −50 mV before being stepped from −120 mV to +80 mV in 20 mV increments when recording. The step duration was 2 s and at least 3 recordings were taken from each cell. Recordings were sampled at 20 kHz and filtered online at 10 kHz. The intracellular solution (317.6 osmol) contained 20 mM of CsCl, 10 mM of EGTA, 7.2 mM of CaCl_2_, 2 mM of MgCl_2_, 10 mM of HEPES, 10 mM of glucose, and 110 mM of Cs aspartate, adjusted to pH 7.2 with CsOH. The extracellular solution (339 osmol) contained 140 mM of NaCl, 2 mM of CaCl_2_, 1 mM of MgCl_2_, 10 mM of HEPES, 10 mM of glucose and 30 mM of mannitol, adjusted to pH 7.4 with NaOH. The low Cl^−^ extracellular solution (339 osmol) contained 140 mM of sodium aspartate, 2 mM of CaCl_2_, 1 mM of MgCl_2_, 10 mM of HEPES, 10 mM of glucose and 30 mM of mannitol, adjusted to pH 7.4 with NaOH. Cells showing a GFP signal were used for assessment of current magnitudes. Regarding 4,4′-Diisothiocyano-2,2′-stilbenedisulfonic acid (DIDS) (53005-05-3, EMD Millipore Corp., Burlington, MA, USA), 1 mM was dissolved in DMSO and added to extracellular bath solution before relevant patch-clamp experiments.

### 4.6. Data Analysis

Quantitative data collected from at least three separate experiments were plotted as means ± standard error of the mean (±s.e.m.). Statistical analysis was performed by one-way ANOVA or Student’s t test using GraphPad Prism version 9.1.2.

## Data Availability

The datasets used and analysed during the current study are available from the corresponding author on reasonable request. ZINC DrugBank FDA drug library available from https://zinc.docking.org/catalogs/dbfda/ (accessed on 4 January 2023).

## Supplementary Materials

The following supporting information can be downloaded at: https://www.mdpi.com/article/10.3390/molecules28083317/s1, Figure S1: Validation of COPII docking model; Figure S2: Effect of 4PBA on Cl^−^ currents recorded in HEK293T cells by whole-cell patch-clamp experiments; Figure S3: Effect of 4PBA on Cl^−^ currents recorded in HEK293T cells by whole-cell patch-clamp experiments; Figure S4: Representative whole cell current responses of HEK293T cells transiently transfected with M325T Best1 treated with DMSO or 25 µM tadalafil; Figure S5: Representative whole cell current responses of HEK293T cells transiently transfected with M325T Best1 treated with compounds. References [19,22,26,51,55] are cited in the Supplementary Materials.
