# Supplementary material for: Tadalafil Rescues the p.M325T Mutant of Best1 Chloride Channel
Source: Molecules. 2023 Apr 8;28(8):3317. doi: 10.3390/molecules28083317 (PMC10142963; doi:10.3390/molecules28083317)
Supplement: Supplementary file 1 [file molecules-28-03317-s001.zip › molecules-2279631-supplementary--proofreading_KE.pdf]

Supplementary Materials

S1: Validation of COPII docking model

4PBA was re-docked into the Sec24a binding site (pdb 5vnl) as the carboxylate anion [1] (Figure S1). The ligand was shown to dock in close agreement with the original 4PBA binding pose in the crystal structure (pdb 5vnl), with a root-mean-square deviation (RMSD) value of 2.13 Å.

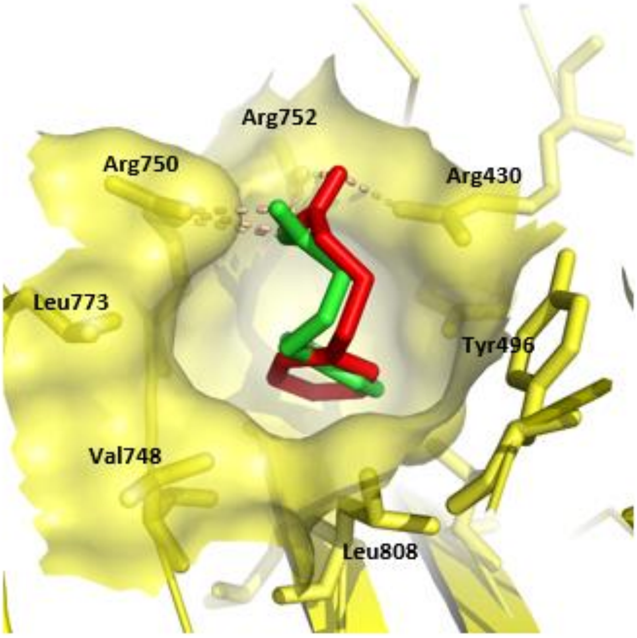

Figure S1. Validation of COPII docking model. The 4PBA ligand (green) present in the Sec24a binding site of the COPII crystal structure (pdb 5vnl) is shown. The 4-PBA conformer obtained from docking studies (red) is overlaid. The close juxtaposition of the two ligands validates the docking model. Hydrogen bonding interactions are indicated between the ligands and residues by dashed lines. Figures generated from pdb file 5vnl using PyMOL software, docking performed with AutoDock Vina software [1–3];

As the RMSD value was > 2.0 Å, the cut-off below which docked ligands are considered 'near-native' relative to the native ligand pose, electron density maps of the COPII binding site were examined using Coot software examined using Coot software (Figure S2). [4,5].

The Fo-Fc map is represented by red or green mesh and represents where the model has over fit or not accounted for the electron density respectively whereas the 2Fo-Fc map includes the Fo-Fc map and electron density around the model (blue mesh).

Figure S2A showed little electron density surrounding the 4PBA ligand for the crystal structure with 1 mM 4PBA, with the pdb file 5vnl used to generate the original docking model. Therefore, a second file was used to examine the electron density for the ligand 5vnn with a 4PBA concentration of 50 mM. Figure S2B showed greater electron density for the ligand structure, however there was less electron density visible for the binding site residues, meaning a combination of both 5vnn and 5vnl were used to evaluate the accuracy of the docking model.

Figure S2C, D showed the electron density of the ligand from the 5vnn map viewed in PyMOL. The conformers of the crystal structure ligand (green) and docked ligand (pink) were superimposed. It can be seen that the conformer of 4PBA generated from the docking model gives a better fit to the electron density for the benzene ring (Figure S2D) than the conformer of the 4PBA from the crystal structure (Figure S2C). It was concluded that the conformer of 4PBA generated with the docking model had a similar degree of overlap with the electron density file as the 4PBA ligand in the crystal structure, thus the model was accurate enough to use in further docking experiments.

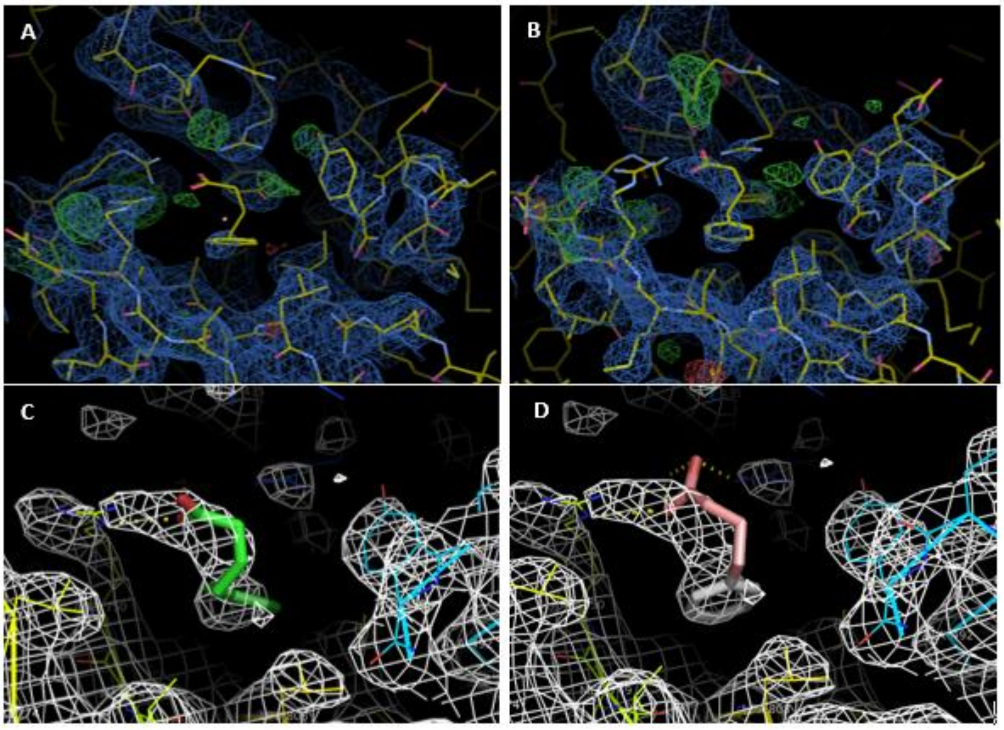

Figure S2. Electron density maps of 4PBA in the COPII binding site. (**A, B**) Electron density of pdb files 5vnl and 5vnn respectively viewed using Coot software [1,4]. The Fo-Fc map is represented by red (over fit of model) or green mesh (unaccounted for by model), the 2Fo-Fc map is represented by blue mesh, viewed at 1.4 RMSD; (**C, D**) Electron density overlaid with conformers of 4PBA from crystal structure and docking model. (**C**) Conformer of ligand from crystal structure. (**D**) Conformer of ligand from docking model, 2Fo-Fc electron density map of pdb 5vnn represented by mesh, viewed using PyMOL software [1,3].

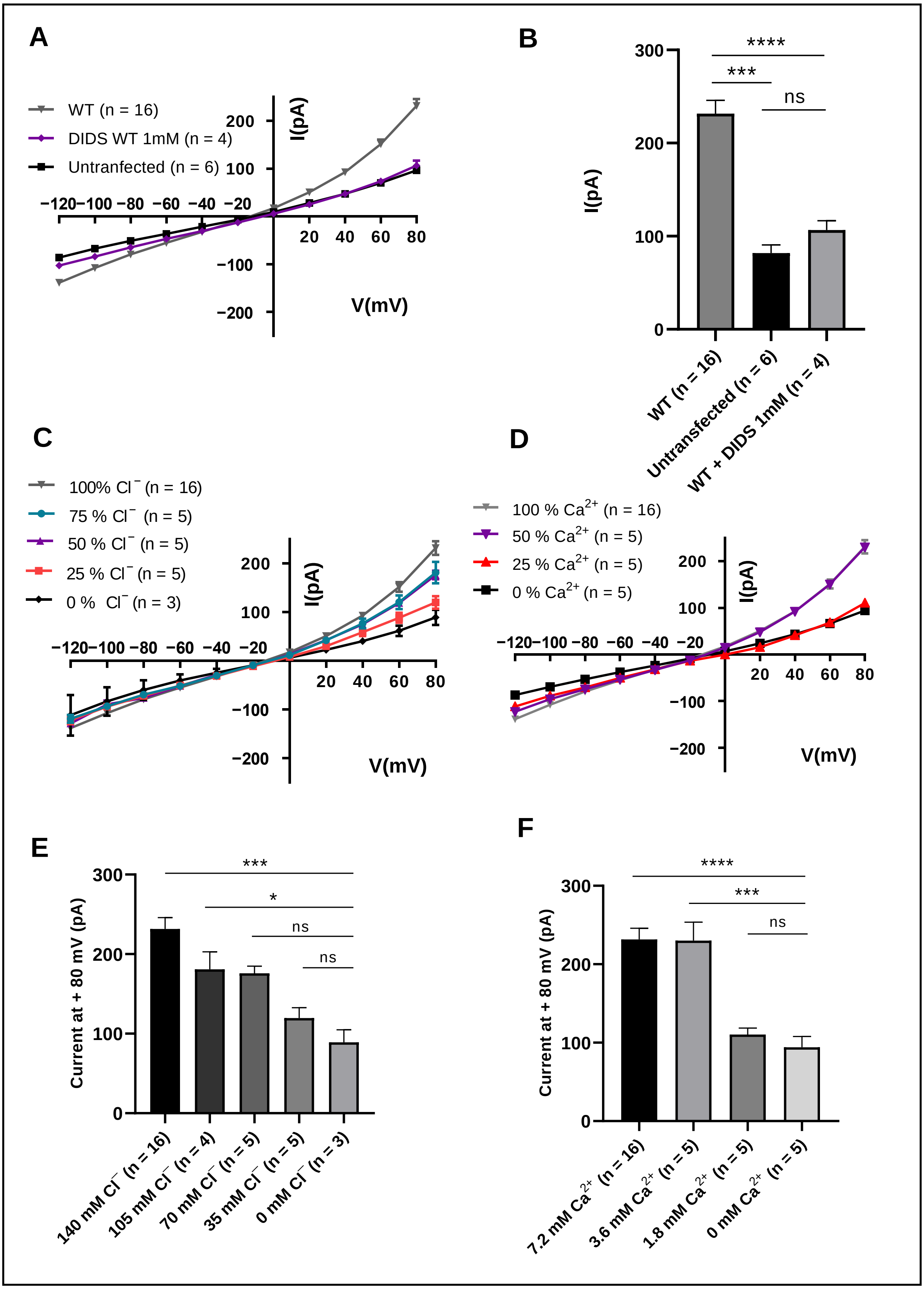

A
WT (n = 16)
DIDS WT 1mM (n = 4)
Untranfected (n = 6)
I(pA)
V(mV)
B
I(pA)
****
***
ns
WT (n = 16)
Untransfected (n = 6)
WT + DIDS 1mM (n = 4)
C
100% Cl⁻ (n = 16)
75 % Cl⁻ (n = 5)
50 % Cl⁻ (n = 5)
25 % Cl⁻ (n = 5)
0 % Cl⁻ (n = 3)
I(pA)
V(mV)
D
100 % Ca2+ (n = 16)
50 % Ca2+ (n = 5)
25 % Ca2+ (n = 5)
0 % Ca2+ (n = 5)
I(pA)
V(mV)
E
Current at + 80 mV (pA)
***
*
ns
ns
140 mM Cl⁻ (n = 16)
105 mM Cl⁻ (n = 4)
70 mM Cl⁻ (n = 5)
35 mM Cl⁻ (n = 5)
0 mM Cl⁻ (n = 3)
F
Current at + 80 mV (pA)
****
***
ns
7.2 mM Ca2+ (n = 16)
3.6 mM Ca2+ (n = 5)
1.8 mM Ca2+ (n = 5)
0 mM Ca2+ (n = 5)

Figure S3. Cl⁻ currents recorded in HEK293T cells by whole-cell patch-clamp experiments. The membrane potential was held at −50 mV and recordings were taken in the range of −120 mV to +80 mV in Δ20 mV steps of 2 s each. (**A**) Mean current/voltage relationships for WT Best1 expressing cells in the presence or absence of 1 mM DIDS, a Cl⁻ channel inhibitor (**B**) Currents measured at + 80 mV (pA) for WT Best1 before and after treatment with 1 mM DIDS. Treatment with DIDS results in a block of WT Cl⁻ conductance, confirming that transfected cells express a Cl⁻ channel; (**C**) Mean current/voltage relationships for WT Best1 expressing cells treated with decreasing concentrations of Cl⁻ in extracellular buffer, (**E**) Currents measured at + 80 mV (pA). The mean current decreases proportionally with Cl⁻ concentration, confirming that cells express a Cl⁻ conducting channel; (**D**) Mean current/voltage relationships for WT Best1 expressing cells treated with decreasing concentrations of Ca^{2+} in intracellular buffer, (**F**) Currents measured at + 80 mV (pA). The mean current is unchanged at 50 % Ca^{2+} but drops significantly at 25 and 0 % Ca^{2+}, confirming that cells express a Ca^{2+} dependent channel and demonstrating that the minimum amount in the intracellular buffer required for channel activation is 50 % Ca^{2+} (3.6 mM); Results presented as mean ± s.e.m, **p*<0.05, ****p*<0.001, *****p*<0.0001, as calculated by one-way ANOVA.

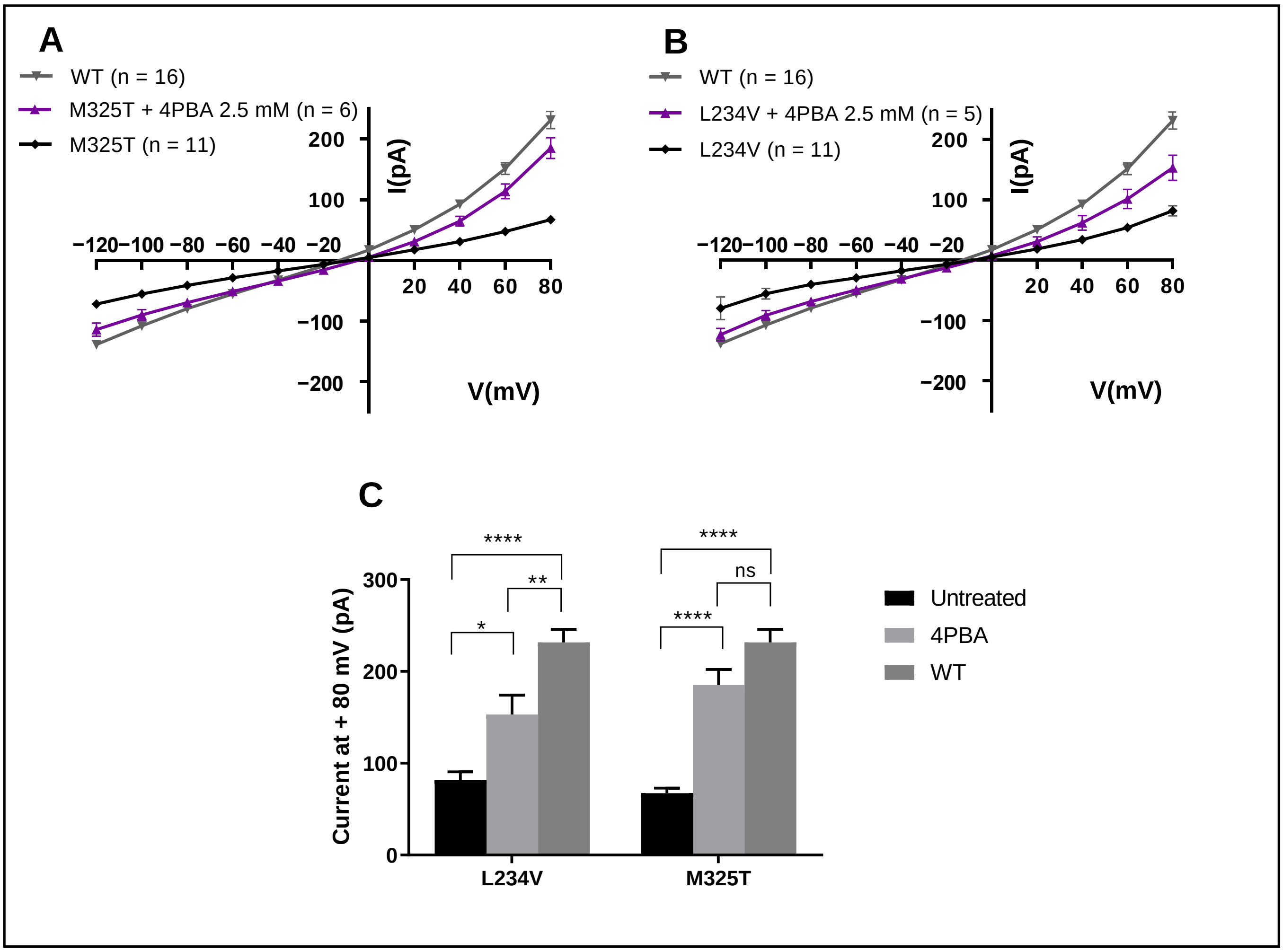

Figure S4. Effect of 4PBA on Cl⁻ currents recorded in HEK293T cells by whole-cell patch-clamp experiments. The membrane potential was held at −50 mV and recordings were taken in the range of −120 mV to +80 mV in Δ20 mV steps of 2 s each. (**A, B**). Mean current/voltage relationships for mutant Best1 expressing cells

{M325T (ARB) and L234V (BVMD)} before and after treatment with 2.5 mM 4PBA. (**C**) Currents measured at + 80 mV (pA). Addition of 4PBA results in rescue of p.M325T or p.L234V Cl^- current to 80 or 66 % of WT Cl^- conductance levels respectively (Results presented as mean ± s.e.m, *p<0.05, **p<0.01, ****p<0.001, as calculated by one-way ANOVA).

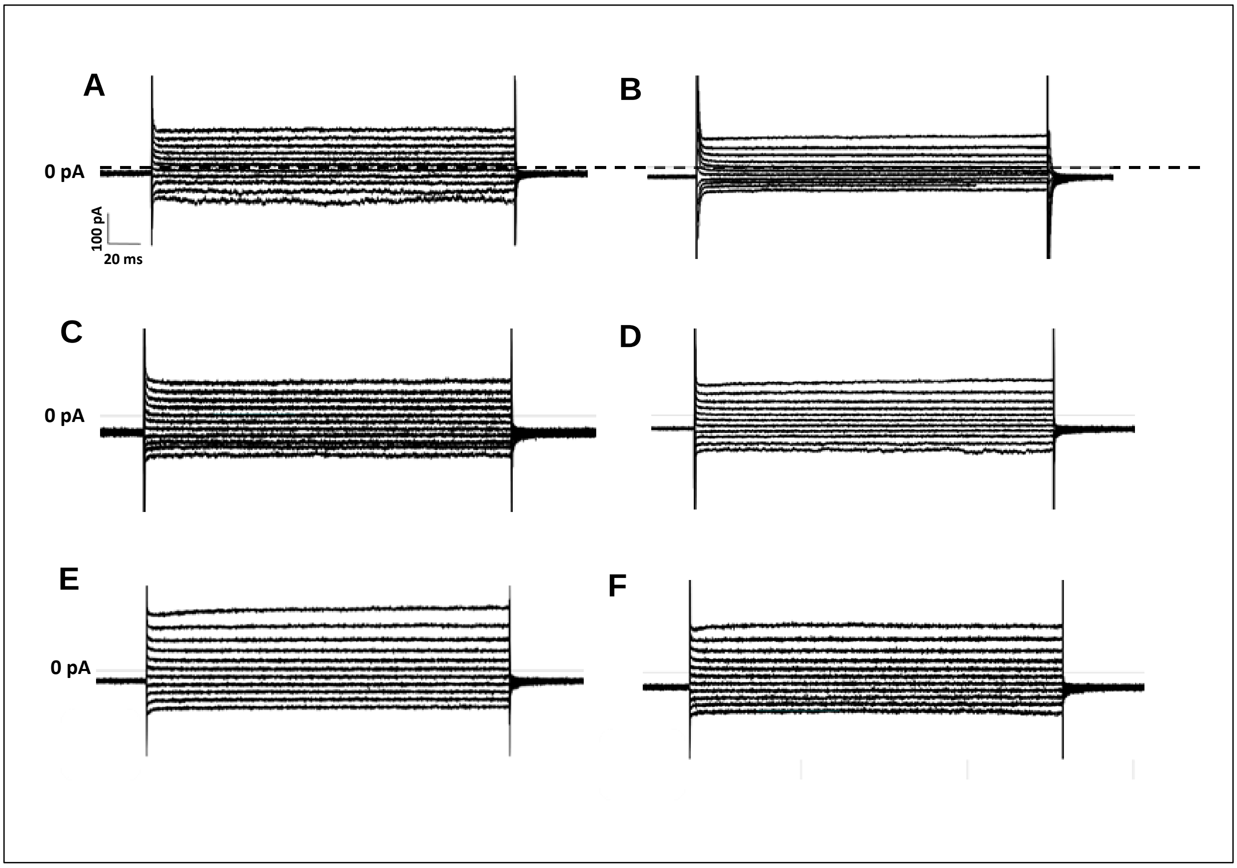

Figure S5. Representative whole cell current responses of HEK293T cells transiently transfected with M325T Best1 treated with (**A**) 0.001 % DMSO; (**B**) 25 μM risperdal in DMSO; (**C**) 25 μM thalidomide in DMSO; (**D**) 25 μM paliperidone in DMSO; (**E**) 25 μM tadalafil in DMSO; (**F**) 2.5 mM 4PBA in water. The membrane potential was held at −50 mV and recordings were taken in the range of −120 mV to +80 mV in Δ20 mV steps of 2 s each.

References:

1. Ma, W.; Goldberg, E.; Goldberg, J. ER retention is imposed by COPII protein sorting and attenuated by 4-phenylbutyrate. *Elife* **2017**, *6*, e26624. https://doi.org/10.7554/eLife.26624.

2. Trott, O.; Olson, A.J. AutoDock Vina: Improving the speed and accuracy of docking with a new scoring function, efficient optimization, and multithreading. *J. Comput. Chem.* **2010**, *31*, 455–461. https://doi.org/10.1002/jcc.21334.

3. Schrödinger, L. *The PyMOL Molecular Graphics System*; Version 1.3; DeLano scientific: San Carlos, CA, USA, 2010.

4. Emsley, P.; Lohkamp, B.; Scott, W.G.; Cowtan, K. Features and development of Coot. *Acta Crystallographica. Sect. D Biol. Crystallogr.* **2010**, *66*, 486–501. https://doi.org/10.1107/s0907444910007493.

5. Bell, E.W.; Zhang, Y. DockRMSD: An open-source tool for atom mapping and RMSD calculation of symmetric molecules through graph isomorphism. *J. Cheminform.* **2019**, *11*, 40. https://doi.org/10.1186/s13321-019-0362-7.
